# Identifying clinically relevant findings in breast cancer using deep learning and feature attribution on local views from high-resolution mammography

**DOI:** 10.3389/fonc.2025.1601929

**Published:** 2025-09-23

**Authors:** Diego Mellado, Leondry Mayeta-Revilla, Julio Sotelo, Marvin Querales, Eduardo Godoy, Scarlett Lever, Fabian Pardo, Steren Chabert, Rodrigo Salas

**Affiliations:** ^1^ PhD. Program in Health Sciences and Engineering, Universidad de Valparaíso, Valparaíso, Chile; ^2^ Center of Interdisciplinary Biomedical and Engineering Research for Health MEDING, Valparaíso, Chile; ^3^ Instituto de tecnología para la Innovación en Salud y Bienestar (ITISB), Facultad de Ingeniería, Universidad Andres Bello, Viña del Mar, Chile; ^4^ Millenium Institute for Intelligent Healthcare Engineering - iHealth, Santiago, Chile; ^5^ Departamento de Informática, Universidad Técnica Federico Santa María, Santiago, Chile; ^6^ School of Medical Technology, Universidad de Valparaíso, Viña del Mar, Chile; ^7^ Informatics Engineering School, Universidad de Valparaíso, Valparaíso, Chile; ^8^ School of Biomedical Engineering, Universidad de Valparaíso, Valparaíso, Chile; ^9^ School of Medicine, Universidad de Valparaíso, Viña del Mar, Chile; ^10^ Metabolic Diseases Research Laboratory (MDRL), Universidad de Valparaíso, San Felipe, Chile

**Keywords:** breast cancer, deep learning, explainable artificial intelligence, feature attribution, mammography, medical image analysis

## Abstract

**Introduction:**

Early detection of breast cancer via mammography screening is essential to improve survival outcomes, particularly in low-resource settings such as the global south where diagnostic accessibility remains limited. Although Deep Neural Network (DNN) models have demonstrated high accuracy in breast cancer detection, their clinical adoption is impeded by a lack of interpretability.

**Methods:**

To address this challenge, CorRELAX is proposed as an interpretable algorithm designed to quantify the relevance of localized regions within high-resolution mammographic images. CorRELAX evaluates the contribution of partial local information to the model’s global decision-making and computes correlations between intermediate feature representations and predictions to produce global heatmaps for lesion localization. The framework utilizes a DNN trained on multi-scale crops of annotated lesions to effectively capture a spectrum of lesion sizes.

**Results:**

Evaluation on the VinDr-Mammo dataset yielded F1 Scores of 0.8432 for calcifications and 0.7392 for masses. Heatmap localization accuracy was assessed using the Pointing Game metric, with CorRELAX achieving average accuracies of 0.6358 based on model predictions and 0.5602 using the correlation maps, indicating robust lesion localization capabilities.

**Discussion:**

These results demonstrate that CorRELAX generates interpretable coarse-segmentation maps that enhance automated lesion detection in mammography. The improved interpretability facilitates clinically reliable decision-making and addresses a critical barrier toward the integration of AI-based methods in breast cancer screening workflows.

## 1 Introduction

Breast Cancer is the most common form of cancer among women worldwide (1). In Latin America, multiple economic, geographical, and cultural barriers limit access to screening procedures, medical resources for diagnosis, and clinical research, leading to lower regional survival outcomes (2–4). In Chile, similar trends are also observed, as patients with access to private healthcare and who reside in central urban areas have higher survival rates than those using the public healthcare system and living in other regions of the country (5). Early detection of breast cancer using mammography has shown a significant reduction of 20% in breast cancer mortality risk, according to the World Health Organization (6). Furthermore, access to screening procedures has improved prognosis and survival rates for patients in both public and private healthcare systems (7).

In recent years, the integration of Deep Learning (DL) and other Machine Learning (ML) techniques as diagnostic assistance tools has increased, reporting an increase in accuracy and improved efficiency in comparison to traditional computer-assisted systems (8–10) It has also been shown to improve sensitivity, reduce false negatives in malignancy detection, particularly for junior radiologists (11), and reduce intra-reader variability when used for breast lesion assessment on multi-modal studies sources, such as breast ultrasound and tomosynthesis (12, 13). However, integrating these ML models into the radiologist’s diagnosis workflow faces challenges due to the lack of human-interpretable explanations of their decision process. Explainable Artificial Intelligence (XAI) has emerged as a tool for addressing biases present in these models, clarifying the relationship between input and reported predictions, and enabling more transparent and informed choices while ensuring that medical personnel remain in the loop (14, 15).

Feature Attribution methods attempt to assign an importance score to a model’s input features by decomposing each feature’s effect on the resulting output, thereby identifying which feature most influences the model’s decision function. Gradient-based feature attribution methods are commonly used to provide interpretability to black-box models by visualizing how input features contribute to their inference, measuring how gradients are affected within the model, and presenting using saliency maps (16). Grad-CAM and its variants, which generate visual explanations by highlighting critical regions from the input image, based on gradient information from a particular label from the output, towards the inspected internal layer of the model (17) Other feature attribution methods include LIME (18), which approximates the model’s decision boundary using a simpler, more shallow model to provide local explanations from a particular feature. More recent approaches include methods involving occlusion of features, such as RISE (19) and RELAX (20), which mask regions from the input image to create feature importance maps assigned based on the changes in prediction using masked information. The latter extends this concept by quantifying a feature’s importance and uncertainty through the comparison of changes in internal feature vectors between masked inputs and the original input.

A key limitation when using DL models is their dependence on the input resolution to extract information. In medical imaging, input images are often down-scaled to lower resolutions (such as 224 × 224 pixels on standard models) when using these models. Due to image compression, smaller lesions and other clinically relevant findings, such as micro-calcifications, can be missed when inspected by these and not considered in the final prediction. Additionally, by design, most feature attribution methods cannot present the interaction between local elements and show the relationships between similar features across different regions within the input image. Moreover, accurately generating automatic segmentation maps can be challenging due to the variability in lesion sizes and the ambiguity of tissue boundaries. Yet, these can assist radiologists in locating less conspicuous lesions that automatic methods would otherwise ignore. These limitations underscore the importance of using high-resolution inputs and techniques to capture and evaluate all relevant information accurately during inference.

To address these challenges, this work proposes a method for evaluating the contribution of local information in high-resolution mammography images to the decision-making process of deep learning models. The proposed approach employs a sliding-window strategy to extract internal feature representations and the resulting predictions from small regions across the image. And measure the correlation between the similarity distances of partial representations and those of their corresponding unmasked windows. These measurements are then combined into a global prediction map, representing the likelihood of pathological findings. A correlation map, which serves as a visualization tool to indicate how similar local features are to the model’s learned knowledge. The proposed method and the resulting maps provide an interpretable representation of the model decision-making process and allow for obtaining a coarse segmentation of potential lesions, enhancing the detection of smaller findings that might be missed with traditional down-sampled approaches.

The core novelty of this work lies in the introduction of CorRELAX, a correlation-based feature attribution method designed for the local assessment of image regions in mammography. In contrast to existing explainability methods that often rely on global saliency or gradient-based responses, CorRELAX quantifies the alignment between internal feature representations and output predictions under partial, random occlusions. This approach provides a robust explanation of how incomplete yet informative regions support the model’s inference, even when pathological findings are underrepresented in the data. Additionally, the use of sliding windows for attribution remains an underexplored strategy for explainable artificial intelligence methods applied to medical imaging. By combining local prediction maps and correlation heatmaps, CorRELAX provides coarse but interpretable segmentation maps of lesions present within mammography images, facilitating their localization.

This paper is organized as follows: In section 2, a review of the literature on the application of XAI algorithms is presented, and more specifically, Feature Attribution Methods to provide explainability to Convolutional Neural Networks and their applications to mammography imaging. Section 3 describes the Dataset used for training, our DL model used for this task, and the proposed algorithm. An outline of the training procedure and evaluation is provided, along with the tests used to measure the precision and stability of our interpretable algorithm. Section 4 shows the results of the proposed experiment, using both a validation sample from the training dataset and examinations from Chilean patients. Finally, section 5 presents a discussion regarding our algorithm’s performance compared to similar experiments, limitations of the presented research, and potential future work to improve the evaluation strategy.

## 2 Related work

In recent years, multiple approaches have been proposed to provide explainability to ML models for breast anomaly detection. In a previous work, the use of large language models for identifying possible findings annotated in mammography reports and the effects of laterality when reporting these findings (21, 22) was evaluated. Globally, recent studies have primarily focused on extracting interpretable features from mammography images that provide insights into the location and characteristics of breast lesions, as well as the importance of neighboring regions in the image for accurate diagnosis.

### 2.1 Saliency maps for breast lesion detection

Saliency maps, particularly Grad-CAM-based methods (17), are widely used for breast lesion detection and localization in mammography. These methods, easily integrated into DL models, use gradient information to generate heatmaps highlighting the most relevant regions on input images. Their ability to identify Regions of Interest (ROIs) makes them a popular choice for providing interpretability in mammography lesion detection.

For instance, Farrag et al. (23) proposed an XAI system for mammogram tumor segmentation using double-dilated convolutions to mitigate local spatial resolution loss and employing Grad-CAM and occlusion sensitivity to identify regions containing masses. Similarly, Dahl et al. (24) proposed a two-stage analysis pipeline using a *ResNet-121* architecture to obtain a holistic risk score of the entire mammography image. Grad-CAM was used to identify the ROI for potential malignancies and refined at a second stage to extract a detailed heatmap at the location. Lou et al. (25) developed a Multi-level Global-guided Branch-attention Network (MBGN) for mass classification in mammography, employing Grad-CAM to validate the relation of the selected features to the ground truth. Likewise, Al-Tam et al. (26) proposed a multi-modal breast cancer detection framework that combines mammography and ultrasound images. Using a *YOLOv8* architecture for ROI detection, a DL ensemble model for malignancy classification, and Grad-CAM for feature visualization of the ROI, providing contextual information of the detected lesions.

On the other hand, Pertuz et al. (27) evaluated different pre-trained DL architectures for breast lesion detection by comparing their saliency maps obtained using Grad-CAM with manual segmentations by radiologists. Their findings revealed a low overlap between the identified saliencies and annotations, suggesting that these models rely upon general features rather than specific elements for classifying malignancies. Similarly, Mobini et al. (28) studied multiple DL architectures using Grad-CAM++ (29), a generalized variant of Grad-CAM that uses a Rectified Gradient to detect breast arterial calcifications in mammography images. Their research highlighted that simpler models, such as *VGG16* and *MobileNet*, outperformed more complex architectures in terms of classification accuracy and the quality of saliency maps.

### 2.2 Comparisons between explainable methods for breast lesion detection

While Grad-CAM remains a widely popular method for generating visual explanations, its limitations have prompted comparisons to similar techniques. A drawback of this method is its tendency to generalize over broader regions of the input image, leading to a loss of detail that can impact the precision of saliency maps, particularly for smaller lesions and calcifications. This has motivated researchers to explore alternatives to the relationship between input and predictions. For instance, Ahmed et al. (30) compared explanations generated by different XAI methods, including LIME, SHAP, and Grad-CAM, across various DL architectures such as *VGG16*, *Inception-V3*, and *ResNet*. Compared with annotations from the CBIS-DDSM dataset, their analysis highlighted differences in performance when aligned with their explanations. Similarly, Barnett et al. (31) proposed an ML-based system that compares information from input images with prototypical examples from training data as case-based explanations. This similarity measurement is then used to classify breast mass margins, obtaining a measurement of malignancy. This measurement is then added to the final lesion prediction, and their explanations are compared to Grad-CAM and Grad-CAM++.

Additionally, Rafferty et al. (32) evaluated methods such as LIME, SHAP, and RISE to identify regions for breast cancer malignancy classification. They noted that these methods have low agreement with the radiologists’ evaluations of lesion relevance. While RISE provided marginally better explanations, none of these methods accurately highlighted the precise region, showing the limitations of these methods on this task. In contrast, Ortega-Martorell et al. (33) proposed a method based on Fisher Information Networks (FIN) to visualize and quantify similarities between learned features. Their approach provides insights into the characteristics and similarities of a particular lesion, as well as its resemblance to learned features, describing specific elements in both benign and malignant masses and calcifications.

Gerbasi et al. (34) developed a DL pipeline for segmentation and malignancy classification of microcalcifications within mammography images. Using a UNet for semantic segmentation of clusters of calcifications within patches of fixed size from mammography images. Followed by the classification of these clusters using a *ResNet-18* architecture fine-tuned for malignancy classification. Additionally, the classifier is later inspected using Grad-CAM and SHAP to identify local regions within these clusters that indicate a malignancy association within the image, providing explanations for the resulting prediction.

Prodan et al. (35) compared multiple DL classifiers based on both CNN and Vision Transformers (ViT), for a malignancy classification task using mammography images and using saliency methods to highlight areas of importance for the classifier for its decision-making process. To reduce imbalance within their training data, they applied a Style-GAN XL (36) to generate positive samples similar to those present in the dataset. Each image was then evaluated using Grad-CAM, which highlighted the regions that had the most impact on the classification task and drew a bounding box around the location of any potential lesions present.

Prinzi et al. (37) introduced *Rad4XCNN*, a *post-hoc*, model-agnostic method for global explanation of CNN models applied to a malignancy classification task of breast ultrasound images. This method aims to enhance the interpretability of CNN-derived features from different *ResNet*, *DenseNet*, and *ViT* architectures by quantifying their correlation with clinically meaningful radiomic features using Spearman’s rank correlation. By identifying deep features with strong correlations to radiomic descriptors, this method enables the construction of class-independent, global explanations aligned with established clinical knowledge. The authors evaluated their method on breast ultrasound images from a publicly available dataset for pre-training, and two in-house datasets from different clinical centers for internal and external validation. While CNN architectures, such as *ResNet* and *DenseNet*, demonstrated robust predictive performance and yielded higher correlations with radiomic features, *ViT*-derived features showed no meaningful alignment. The authors also compared their method to local saliency map explanation methods, such as Grad-CAM, Eigen-CAM, and Score-CAM. These produced visually inconsistent explanations, particularly for misclassified samples, compared to their proposed method.

### 2.3 Impact of input image resolution

A present challenge for lesion detection in mammography lies in the impact of the resolution of the input images used for DL models, which hampers the detection of smaller lesions like calcifications. Most models down-sample the input images to a predefined resolution, often losing relevant information from smaller-sized elements. Conversely, high-resolution inputs can improve detection but significantly increase computational requirements for training and inference.

Several studies have proposed strategies to address this trade-off. Farrag et al. (23), for example, utilized double-dilated convolutions to improve segmentation accuracy but down-scaled images to 512×512 pixels. Similarly, Dahl et al. (24) used a two-stage approach, down-sampled the image to a 976 × 976 resolution to improve the detection of smaller lesions before rescaling the image further to 512 × 512 in their second stage to extract interpretable features from the identified ROI from the first stage. Meanwhile, Al-Tam et al. (26) rescaled the input to 640×640 for their object detection stage and later downsampled to 128 × 128 pixels during runtime training of ROI areas. For calcification detection, Mobini et al. (28) scaled input images to 1576 × 768 across their evaluated models to ensure their models’ response to the smaller size of these lesions.

Despite these efforts, most studies rely on smaller input resolutions, ranging between 224 × 224 to 512 × 512. While often sufficient for malignancy classification and coarse lesion localization tasks, these resolutions fall short on detection tasks involving smaller-sized elements. This evidences a trade-off that prioritizes computational load at the cost of precision to identify smaller clinically significant features.

## 3 Materials and methods

**Figure 1:**
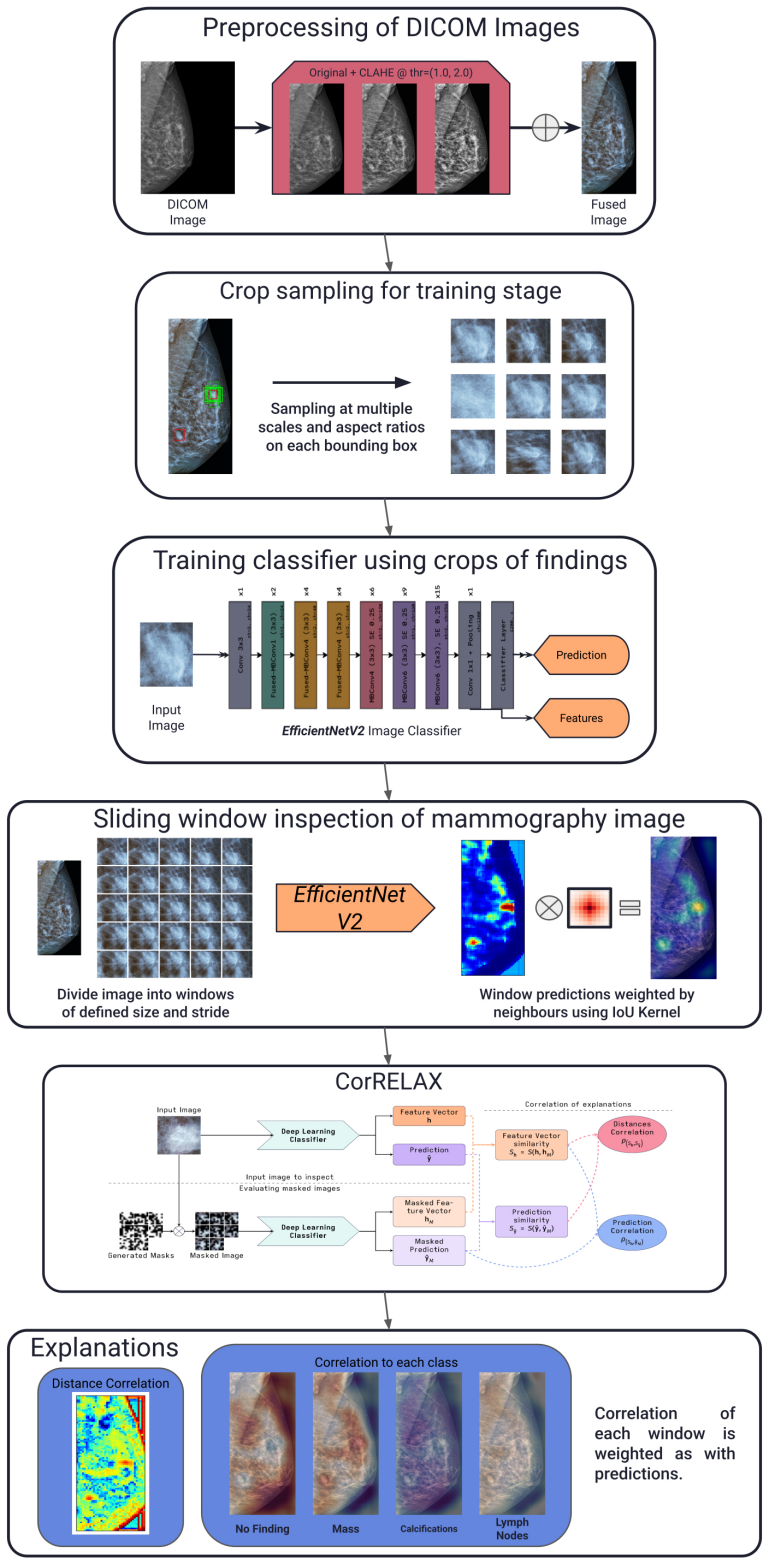
General schema of our proposed pipeline. Describing the preprocessing of each mammography image, sampling of annotated crops for training, reconstruction using sliding windows of the resulting prediction using the trained classifier, the proposed explanation method and examples of the resulting correlation maps obtained.

The proposed method, in summary, studies each view from a mammography image independently. Each image is initially segmented into its corresponding ROI and then divided into small intersecting windows, which are then evaluated using a CNN classifier. Trained using crops of pathological findings annotated from a publicly-available dataset. The model outputs both the multi-label prediction of lesions present within the crop and a feature vector representation of the input image. Both predictions and feature vector representations are compared to the resulting outputs from occluded versions of the input image, yielding a correlation measurement for each window. These values are then combined using our adjacency kernel to reconstruct the final prediction and correlation maps per class, along with a distance map indicating the relevance of each window to the internal learned features within the model. **Figure 1** illustrates the complete pipeline of the proposed method, which is described in the following subsections.

### 3.1 Preprocessing

**Figure 2:**
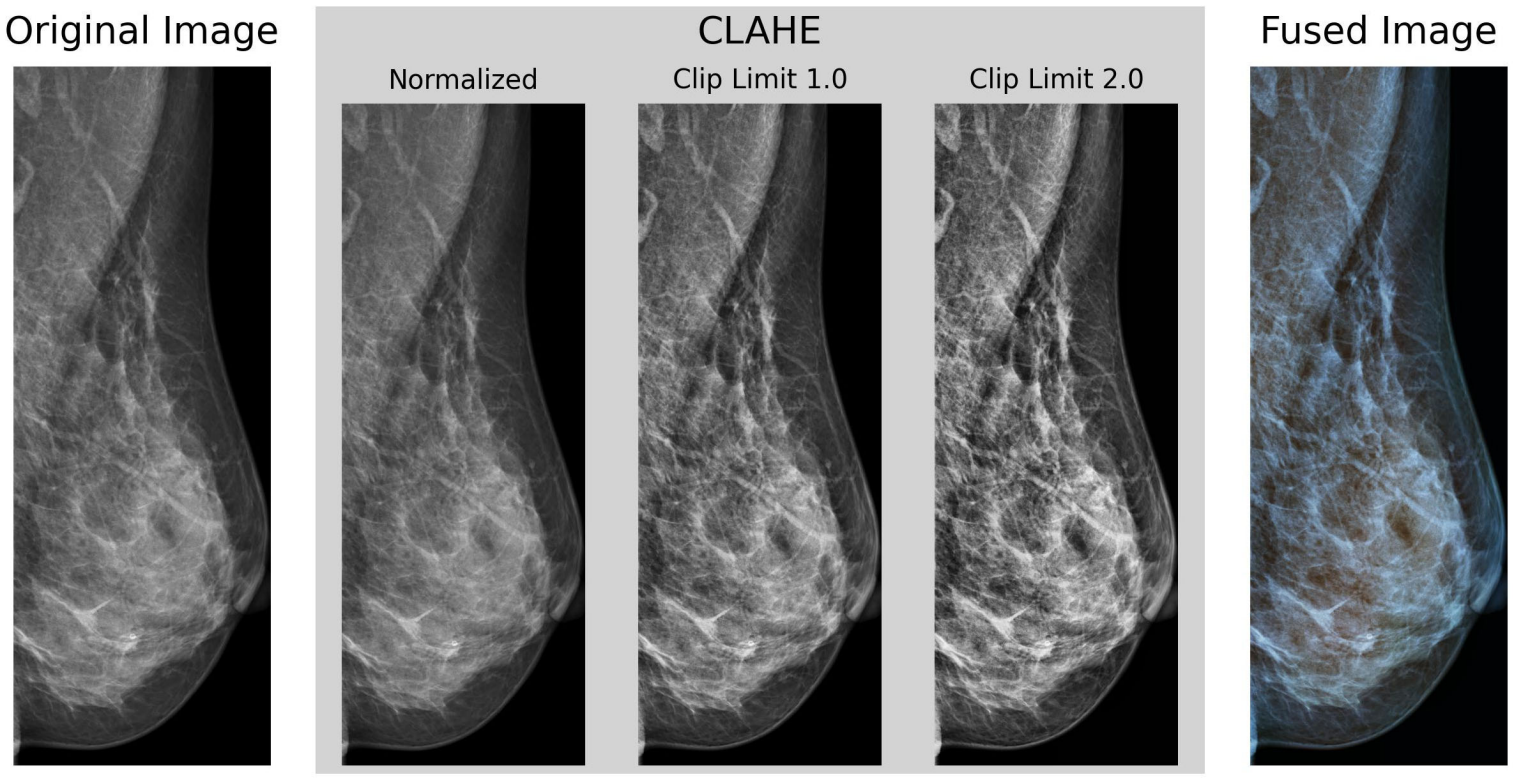
Example of our preprocessing pipeline, cropped to the identified Region of Interest of the breast region, using Otsu’s Thresholding.

For the preprocessing stage, a set of transformations was implemented, similar to those proposed by (38). **Figure 2** shows an example of the preprocessing pipeline as described in this section.

*VinDr-Mammo* contains images with a mean original size of 2647 × 3387 pixels, ranging between 2012–2812 pixels in width and 2812–3580 pixels in height, and intensity values stored in an unsigned 16-bit integer format. Each image was initially scaled in intensity between 0–1 and inverted if the Photometric Interpretation tag on the DICOM metadata was set to MONOCHROME1 to ensure all images have a black background and white foreground.

Then, each underwent a histogram equalization transformation using the Contrast Limited Adaptive Histogram Equalization (*CLAHE*) algorithm (39) to enhance the contrast of the images. *CLAHE* divides the image into a series of tiles of defined size (8 × 8 pixels in our case), then clips each tile’s histogram to a specified contrast limit. Then, each histogram bin is redistributed across all bins, and the Cumulative Distribution Function (CDF) is calculated. After this, the pixel intensity values are then remapped using the CDF. This process is repeated for each tile, enhancing the contrast locally and avoiding the over-amplification of noise in the image. Finally, each tile is rejoined using bilinear interpolation to obtain the contrast-enhanced image without any artifacts. The OpenCV (40) implementation of the *CLAHE* algorithm with clip limits of 1.0 and 2.0 as utilized. The resulting images were then fused channel-wise to the original non-equalized image, obtaining an RGB representative image as output.

Next, the image was cropped to its ROI via Otsu’s thresholding (41) and contour detection to obtain the bounding box of the breast region. Resulting in a set of images cropped to the breast’s ROI, with an average size of (885 ± 190) × (2497 ± 502) pixels.

### 3.2 Data set

For training, initial testing, and benchmarking, the *VinDr-Mammo* dataset (42) was utilized. This publicly available dataset comprises multi-view mammography images from 5000 patients from the Hanoi University Hospital in Vietnam. This dataset provides bounding boxes of the location of ten different types of lesions present within the breast, including masses, calcifications, asymmetries, and architectural distortions. It also provides the BI-RADS score for each marked finding and the patient’s breast density for each view. This dataset was selected because of its detailed annotations of the location of multiple types of findings beyond masses and calcifications, allowing us to inspect the presence of clinically relevant findings at a local level. However, as some available findings are limited, some categories with similar characteristics, such as focal, global, and (general) asymmetries, nipple, and skin retractions, were grouped into general labels (asymmetries and retractions, respectively). The dataset is divided into training and test sets, containing both Cranio-Caudal (CC) and Medio-Lateral Oblique (MLO) views of both breasts for each patient, split in a 80–20% ratio between training and test sets. All splits were performed using a subject-out scheme to reduce possible bias from data from the same patient in different splits.

For training of the proposed classifier model, each annotated bounding box available was treated as an independent sample, considering that multiple bounding boxes could be present in a single image and that each bounding box could contain various types of lesions. As such, this problem was studied as a multi-label classification task, where each bounding box could be labeled with one or many types of lesions. As many images contain no annotated lesions (labeled as *No findings*), a random area of the image was sampled as a negative example for each image in this subset. Ensuring that the model can learn to differentiate between the presence and absence of lesions. **Table 1** summarizes the distribution of the different types of findings present in the dataset on the training and test sets.

**Table 1:** Number of findings per split in *VinDr-Mammo* dataset.

Finding	Train	Test
No Finding	14589	3643
Mass	989	237
Suspicious Calcification	428	115
Asymmetries	313	79
Architectural Distortion	95	24
Suspicious Lymph Node	46	11
Skin Thickening	45	12
Retractions	39	9

For validation with clinical patients, a set of images provided by a local Hospital in Chile was evaluated. These exams correspond to a set of mammography images acquired for breast cancer screening from a population of adult Chilean women, including both CC and MLO views of these patients, plus the examination report evaluated by radiologists from the hospital. These examinations were used in this study with authorization from the Human Research Ethics Committee of Universidad de Valpara´ıso (CEC-UV), which serves as the study’s Institutional Review Board (IRB). For the evaluation, a set of images in which the report indicated the presence of masses and calcifications, as well as their general location within the body, was selected.

### 3.3 Deep convolutional neural network classifier

The proposed experiment involves classifying clinically relevant findings in mammography images using a multi-label classifier. In a previous work (43), the model selection for this task is detailed and summarized as follows.

Initially, we trained a series of deep learning architectures to determine the best model for our task. Comparing the *EfficientNetV2*, *ResNet50*, *Swin Transformer*, *DenseNet121*, *VGG19*, and *MobileNet* architectures with pre-trained weights on the *ImageNet* dataset; using the implementations provided by PyTorch’s *torchvision* library (44). To ensure uniformity between the models, the final classification layer of each model was replaced with with a 2-layer Dense Network with an initial Dropout layer of 0.5rate, a hidden layer of 512 units, and *ReLU* activation, and a final output layer with the number of classes in the dataset and a *Sigmoid* activation function. These parameters were estimated on initial grid search experiments and were kept constant for all models to ensure a fair comparison.

Each model was trained using a subset of the findings present in the dataset, specifically Masses, Calcifications, Asymmetries, and Suspicious Lymph Nodes, as these are the most common findings. In **Table 2**, the resulting *F1-Score* obtained by each model on the dataset’s test set is presented. The *EfficientNetV2* architecture obtained the best performance on the test set, with an average *F1-Score* of 0.727, outperforming the other architectures by a small margin.

**Table 2:** F1 scores for pathological finding classification task using a subset of *VinDr-Mammo* dataset, comparing different deep learning architectures. (43).

Finding	N	DenseNet121	EfficientNetV2	ResNet50	Swin Transformer	VGG19	MobileNet
Mass	237	0.783	0.815	0.742	0.770	0.756	0.708
Suspicious Calcification	115	0.847	0.865	0.860	0.828	0.873	0.828
Asymmetries	79	0.306	0.295	0.200	0.310	0.204	0.324
Suspicious Lymph Node	11	0.667	0.500	0.737	0.370	0.400	0.476
Weighted Average	442	0.712	0.727	0.675	0.693	0.679	0.665

**Figure 3:**
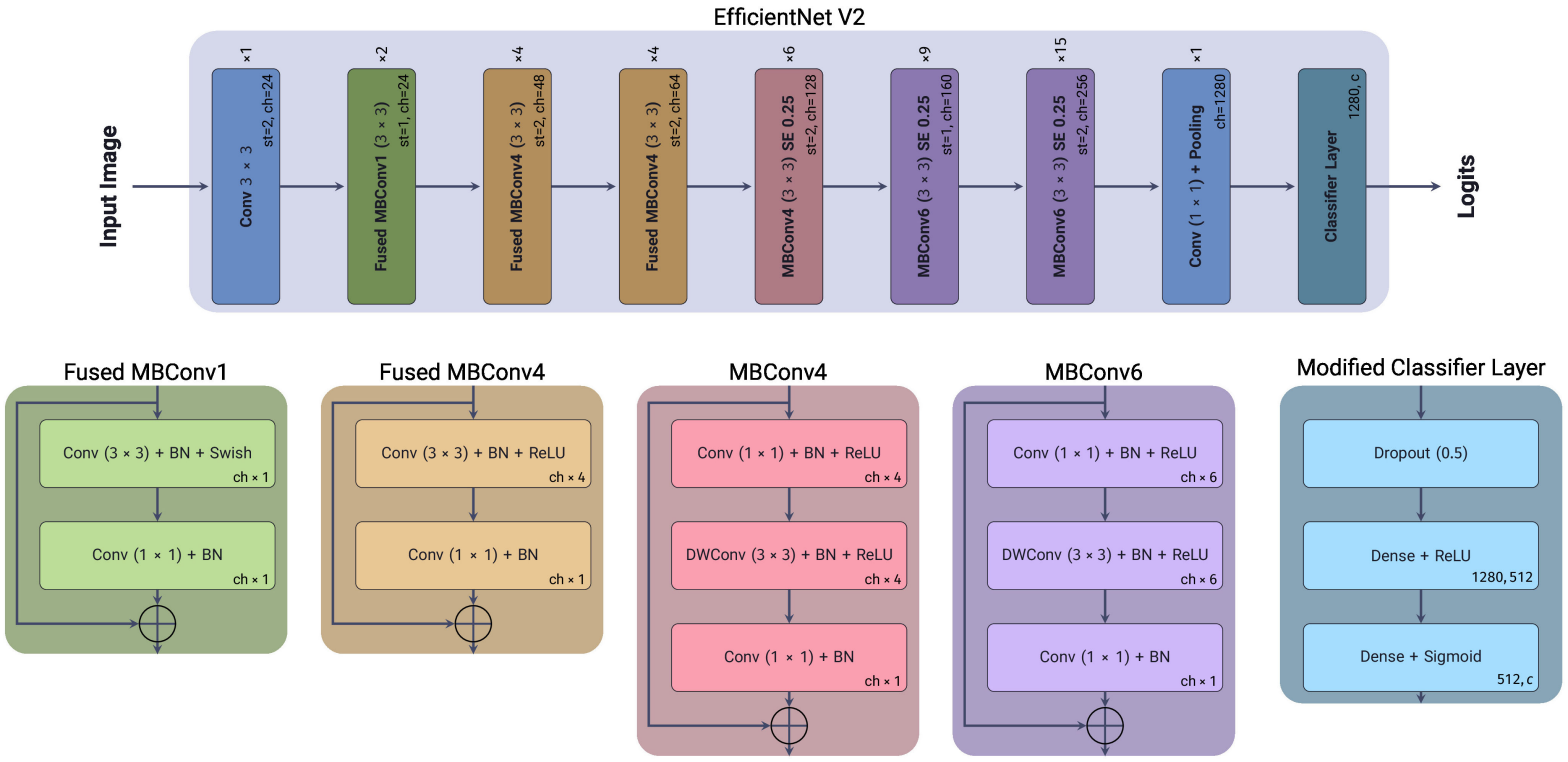
EfficientNetV2 architecture, showing each block’s depth, width, and resolution scaling factors. Included is the modified classification layer used in our experiments.

*EfficientNetV2* (45) is a family of convolutional neural network models optimized for parameter efficiency and computational cost by scaling the depth, width, and resolution of the network in a balanced manner. The original *EfficientNet* architectures were designed to scale the network’s depth, width, and resolution simultaneously on Convolutional Neural Networks (CNNs), using a Neural Architecture Search (NAS) approach to find the optimal scaling factor on each block to balance a trade-off between accuracy and computational cost (46). One of the main innovations of *EfficientNetV2* compared to the original is the replacement of the original MBConv blocks with a new Fused-MBConv block, which combines the original’s depth-wise separable convolution and its expansion convolution into a single operation. Another improvement is using a smaller expansion ratio for the convolutional layers, which reduces the number of parameters required for each layer, and using smaller kernel sizes for the convolutional layers. While compensating for the reduced receptive field by increasing the number of layers in the network. **Figure 3**, shows the architecture of the *EfficientNetV2* model used in our experiments, with our modified classification layer, as previously mentioned.

The model was trained using an *Adam* optimizer (47) with a starting learning rate of 0.001 and a Cosine Annealing decay schedule during 50 epochs, down to a final learning rate of 1 × 10^−7^, using a batch size of 48 samples on an NVidia RTX 4080 GPU. The *Focal Loss* (48) function was employed on optimization. This loss function addresses the effects of extreme class imbalance between positive and negative samples by adding a modulating factor *γ* to the cross-entropy loss, which penalizes the loss of well-classified samples, focusing on the complex examples. As shown in Equation 1, for the case of multi-label classification, it is defined as the logarithm of the predicted probabilities *p* of the ground-truth label vector *y*, modulated by the factor ${\left(1-p\right)}^{\gamma }$ which penalizes errors on complex samples. And an *α* parameter, which acts as a weighting factor between positive and negative labels. When *γ* = 0, the *Focal Loss* is equivalent to the standard cross-entropy loss. Using grid search, the defined parameter values for these were *α* = 0.95 and *γ* = 2.5, as these provided the best performance on the training dataset.


(1) $$\text{FL}\left(p\right)=-\alpha \left[y{\left(1-p\right)}^{\gamma }\log\;\left(p\right)+\left(1-y\right)p^{\gamma }\log\;\left(1-p\right)\right]$$

Multiple data augmentation techniques were applied to the cropped images during the training stage, allowing our model to classify findings across different scales and aspect ratios. On training, the image was cropped using the bounding box annotations of the clinically relevant findings and cropping at different scales (between 0.05–5 times the original bounding box area) and aspect ratios (between 0.33–1.66) from the center of the bounding box. In the case of normal tissue, from each image labeled as *No Finding* a random region was cropped using similar scales and aspect ratios to those used for the positive examples. This cropping was repeated on each training epoch to ensure the diversity of scales for each image.

To further mitigate the impact of the dataset’s class imbalance, each crop was sampled using a Weighted Random Sampling function, where the inverse of the label frequency in the complete dataset determined the weight for each sample. Additionally, a series of transformations was applied to each crop during the training stage. Randomly applying with a probability of 50%, horizontal and/or vertical flips, random rotation between -30° to 30°, and random brightness, contrast, saturation, and hue adjustments. Finally, each crop was resized to a fixed size of 256 × 256 pixels with Bilinear interpolation for the model’s input. During the validation and testing stages, no transformation was applied except for resizing the crops, utilizing the annotated bounding boxes on each sample, and a center crop of the mammography image if the sample was annotated with no findings present.

### 3.4 CorRELAX: correlation of representations for explainability

The proposed method, CorRELAX, is a modification of the RELAX algorithm (20) that expands upon the original method’s measurement of feature importance by measuring the correlation between the distances of representations of the input features and the model’s predictions. This assumes that the distances between feature vectors and predictions of incomplete information of the same input should correlate, as a trained model should infer similar predictions from similar input representations. This correlation should be higher when the model is more confident that the input features are relevant to the prediction, based on the model’s learned knowledge. This method is expected to provide a more robust measurement of importance, as it considers both the expected values of the distances and the uncertainty of the importance of the input features.

**Figure 4:**
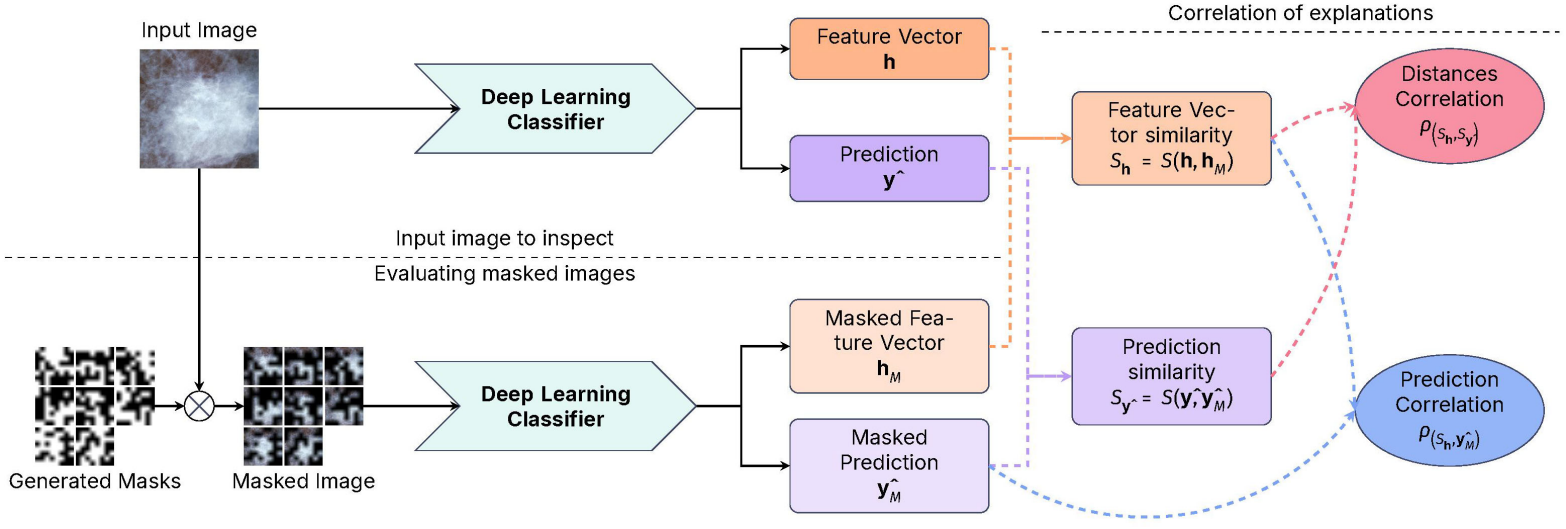
Diagram of the CorRELAX algorithm, showing the method’s workflow for evaluating the correlation between representations and predictions. Using both an input image and random masked versions of itself on a Deep Learning Image Classifier to obtain a metric of the relevance of information from the input to the model’s output, and the correlation between the feature vectors and the predictions.

**Figure 4**, presents a diagram of the workflow of our proposed method. Given an input image $X\in {\mathbb{R}}^{H\times W}$ , of size *H,W*, we do inference using a trained DL model f(X|θ) . Most CNN architectures can be described as two parts:

- A feature extractor *f*_extract_ extracts features at different levels of abstraction from the input image using a series of convolutional and pooling layers.
- A classifier *f*_predict_ takes the extracted features’ representation of the input image and predicts the output class *c* from a Multi-Layer Perceptron (MLP)-like structure with a defined activation function.

Using a set of parameters *θ* learned during training, on inference an internal feature vector of size *D* is extracted from the last layer previous to the classifier stage $h=f_{\text{extract}}\left(X|\theta \right)\in {\mathbb{R}}^{D}$ , and the predicted output from the model $\hat{y}=f_{\text{predict}}\left(h|\theta \right)$ . Following this, a set of random masks M∈ [0, 1]^*b*×*b*^ is created by sampling from a Bernoulli process with a probability *p* of a region being masked, starting from a block of size *b* × *b* which is then up-scaled to the size of the input image, to mask different regions within the input. These masks are then applied, resulting in a set of masked variations of the input image $X_{M}=X\;⊙M$ , which are inputted into the trained model, returning both the masked feature vectors $h_{M}=f_{\text{extract}}\left(X_{M}|\theta \right)$ and the prediction outputs ${\hat{y}}_{M}=f_{\text{predict}}\left(h_{M}|\theta \right)$ from each masked image.

Using a distance function, the similarity between the feature vectors and their masked versions $S_{h}=S_{\left(h,h_{M}\right)}$ and the similarity between the image prediction and the prediction of the masked images $S_{\hat{y}}=S_{\left(\hat{y},{\hat{y}}_{M}\right)}$ is estimated. The *cosine similarity* shown in Equation 2, measures if two vectors are similar in feature space by calculating the cosine of the angle between them. Vectors with similar semantic information will have a cosine similarity closer to 1, while vectors with different information will have a cosine similarity closer to 0.


(2) $$S_{\left(A,B\right)}=\frac{A\cdot B}{\left\|A\|\|B\right\|}$$

Using the estimated distances, two correlation coefficients are then calculated:

- The correlation between the feature vectors’ similarities and the similarities between the model’s predictions $\rho_{\left(S_{h},S_{\hat{y}}\right)}$ .
- The correlation between the feature vectors’ similarities to the probability of the masked views of the input image $\rho_{\left(S_{h},{\hat{y}}_{M}\right)}$ .

The first coefficient $\rho_{\left(S_{h},S_{\hat{y}}\right)}$ , as shown in Equation 3, evaluates the similarity distance between the internal feature vector representations from the original input of the model and the feature vector representation from a set of masked versions of the input image. Then, its correlation to the similarity distance between the resulting predictions from the original input and its masked versions is measured. This results in a value that measures how the model’s learned knowledge aligns with representations and predictions.


(3) $$\rho_{\left(S_{h},S_{\hat{y}}\right)}=\frac{n\sum_{i}S_{h_{i}}S_{{\hat{y}}_{i}}-\sum_{i}S_{h_{i}}\sum_{i}S_{{\hat{y}}_{i}}}{\sqrt{n\sum_{i}S_{h_{i}^{2}}-{\left(\sum_{i}S_{h_{i}}\right)}^{2}}\sqrt{n\sum_{i}S_{{\hat{y}}_{i}^{2}}{\left(\sum_{i}S_{{\hat{y}}_{i}}\right)}^{2}}}$$

The second coefficient $\rho_{\left(S_{h},{\hat{y}}_{M}\right)}$ , shown in Equation 4, evaluates the similarity distance from the internal feature vector representations, and measures its correlation to the probability of belonging to the label *i* ∈ *c* from each masked image output ${\hat{y}}_{M}$ . Obtaining a measure of how partial information at the input impacts the model’s final prediction establishes the importance of the input features to the model’s decision-making process.


(4) $$\rho_{\left(S_{\text{h}},{\hat{y}}_{M}\right)}=\frac{n\sum_{i}S_{{\text{h}}_{i}}{\hat{y}}_{M_{i}}-\sum_{i}S_{{\text{h}}_{i}}\sum_{i}{\hat{y}}_{M_{i}}}{\sqrt{n\sum_{i}S_{{\text{h}}_{i}}^{2}-(\sum_{i}S_{{\text{h}}_{i}}{)}^{2}}\sqrt{n\sum_{i}{\hat{y}}_{M_{i}}^{2}-(\sum_{i}{\hat{y}}_{M_{i}}{)}^{2}}}$$

### 3.5 Experiment

The proposed model was trained to classify cropped samples of mammography images containing clinically relevant findings at various scales and aspect ratios, ensuring adaptability in detecting elements of interest regardless of their size or location. Initially, the classifier was evaluated using cropped samples from the test set corresponding to annotated bounding boxes of findings. Performance metrics, including accuracy, precision, recall, and F1-score, were estimated for each label.

A sliding window approach was applied to the entire mammography image to inspect and identify clinically relevant findings, as previously shown in (43). The mammography image was divided into a set of local views of a defined size and stride. In this experiment, a size of 256 × 256 and a stride of 48 pixels between each window was determined. Each window was input into the model, obtaining the internal feature vector and the multi-label prediction output.

To reconstruct the global prediction, using the projections of all windows within the image, a convolution operation was applied to the prediction of each window, using a kernel that represents the weight of neighboring windows to the current one. This kernel was constructed by estimating the Intersection over the Union (IoU) of the neighboring windows, weighted by the number of overlapping windows for each. The IoU from a pair of rectangular areas (*A,B*) ∈ R^2^ as shown in Equation 10, described each one as a pair of points from the bottom-left corner to the top-right corner of the defined rectangle $A=\left(\left(x_{min}^{A},y_{min}^{A}\right),\left(x_{max}^{A},y_{max}^{A}\right)\right)$ . Equations 5–9 describe each step of the IoU estimation, as follows:


(5) $$A\cap B=\left(\min\;\left(x_{max}^{A},x_{max}^{B}\right)-\max\;\left(x_{min}^{A},x_{min}^{B}\right)\right)$$

(6) $$\cdot \left(\min\;\left(y_{max}^{A},y_{max}^{B}\right)-\max\;\left(y_{min}^{A},y_{min}^{B}\right)\right)$$

(7) $$\text{Area}\left(A\right)=\left(\left(x_{max}^{A}-x_{min}^{A}\right)\cdot \left(y_{max}^{A}-y_{min}^{A}\right)\right)$$

(8) $$\text{Area}\left(B\right)=\left(\left(x_{max}^{B}-x_{min}^{B}\right)\cdot \left(y_{max}^{B}-y_{min}^{B}\right)\right)$$

(9) A∪B=Area(A)+Area(B)−A∩B

(10) $$IoU\left(A,B\right)=\frac{A\cap B}{A\cup B}$$

Each value from the kernel of size $\frac{\text{window}\_\text{size}}{\text{stride}}$ is estimated from the IoU between the center and the neighboring windows with centers at a distance within $\left[\frac{-\text{window}\_\text{size}}{2},\frac{\text{window}\_\text{size}}{2}\right]$ , from the center at each dimension, separated at stride. This kernel is then applied using a 2D-convolution operation to each map. This operation yields a smoothed prediction for each class across a general region of the complete image, taking into consideration how the predictions from each window overlap. As a result, a prediction map was generated for each label, indicating the predicted location of various clinically relevant findings within the mammogram.

A similar approach was used to inspect the image globally using our proposed algorithm. For each window analyzed from a mammography exam, we generated an arbitrarily high number (2560) of masks using an initial mask block size of 8 × 8 pixels and a probability of 0.5 for each region to be masked. All masks were then up-scaled to the original image size using bilinear interpolation and applied to the window in sets of 128 masks for easy computation. The resulting feature vectors and prediction for each mask were accumulated for each window. Then the correlation value of each window’s set of feature vector and prediction is evaluated. Finally, this kernel is applied to each class’s resulting feature relevance metric and correlation maps, obtaining a global heatmap of each label’s feature relevance and correlation.

To evaluate the precision of our method in localizing clinically relevant findings on each mammography image, as reported within the dataset’s bounding boxes, a “Pointing Game” strategy (49) was applied. Given the prediction and feature correlation maps for each label in the dataset, we identified the maximum values for each ground truth label present in each image containing a labeled finding. The location point of these maxima was considered the predicted location of the finding. The “Pointing Game” accuracy for each label was calculated as shown in Equation 11.


(11) $$\text{Accuracy}=\frac{\#\text{Hits}}{\#\text{Hits}+\#\text{Misses}}$$

Considering the effect of the strides between evaluated windows, a prediction was considered a hit if the predicted location fell within the reported bounding box with an offset of 48 pixels within. In the case of the prediction heatmaps, we constrained our evaluation of the maxima to consider a hit if the prediction value was ≥ 25% or ≥ 50%. For the correlation heatmap, a hit was counted if it had a positive correlation within.

Finally, to evaluate the stability of the correlation measurement at lower mask densities, the correlation coefficient on multiple subsets of masks was measured. Starting from an initial arbitrarily large number of generated masks, and reducing the number of applied masks down to a minimum of 128. Then, the distance correlation coefficient was estimated for each subset within each window and compared to the corresponding value obtained from the complete set of generated masks. This allowed us to determine if the correlation distance measure was stable when using fewer masks, providing insights into the robustness of the proposed method under limited conditions.

## 4 Results

### 4.1 Classifier performance on VinDr-Mammo dataset

**Table 3** shows the metrics of the trained classifier on the VinDr-Mammo dataset, evaluated using crops from the clinically relevant findings annotated within the dataset.

**Table 3:** Metrics of our Deep Learning Classifier, trained with crops of pathological findings present in VinDr-Mammo Dataset.

Label	Accuracy	Precision	Recall	F1 Score	Support
No Finding	0.9824	0.9958	0.9844	0.9901	3643
Mass	0.9690	0.7200	0.7595	0.7392	237
Suspicious Calcification	0.9915	0.8846	0.8000	0.8402	115
Asymmetries	0.9790	0.4000	0.1772	0.2456	79
Architectural Distortion	0.9941	0.5000	0.0417	0.0769	24
Suspicious Lymph Node	0.9973	0.5000	0.0909	0.1538	11
Skin Thickening	0.9983	1.0000	0.4167	0.5882	12
Retractions	0.9973	0.3333	0.2222	0.2667	9

The trained model showed a high performance in classifying normal tissue, masses, and suspicious calcifications, achieving an F1-Score of 0.9901, 0.7372, and 0.8402, respectively. The high accuracy in classifying normal tissue can be attributed to its prevalence within the dataset, making our model exceptionally reliable at identifying the absence of findings. The model performs reasonably well for masses and calcifications, considering the challenge of detecting the latter type due to their small size and sparse distribution in mammography images. However, the model struggles with rarer findings (i.e., they have a few limited data samples), such as Architectural distortions, reflecting on their limited representation within the dataset. Similarly, asymmetries also show low performance, likely due to their structural similarity to masses, as asymmetries are defined as an increased density of fibrous gland tissue, resembling masses (50, 51). Particularly at larger window sizes, these become more ill-defined and thus harder to differentiate from masses.

**Figure 5:**
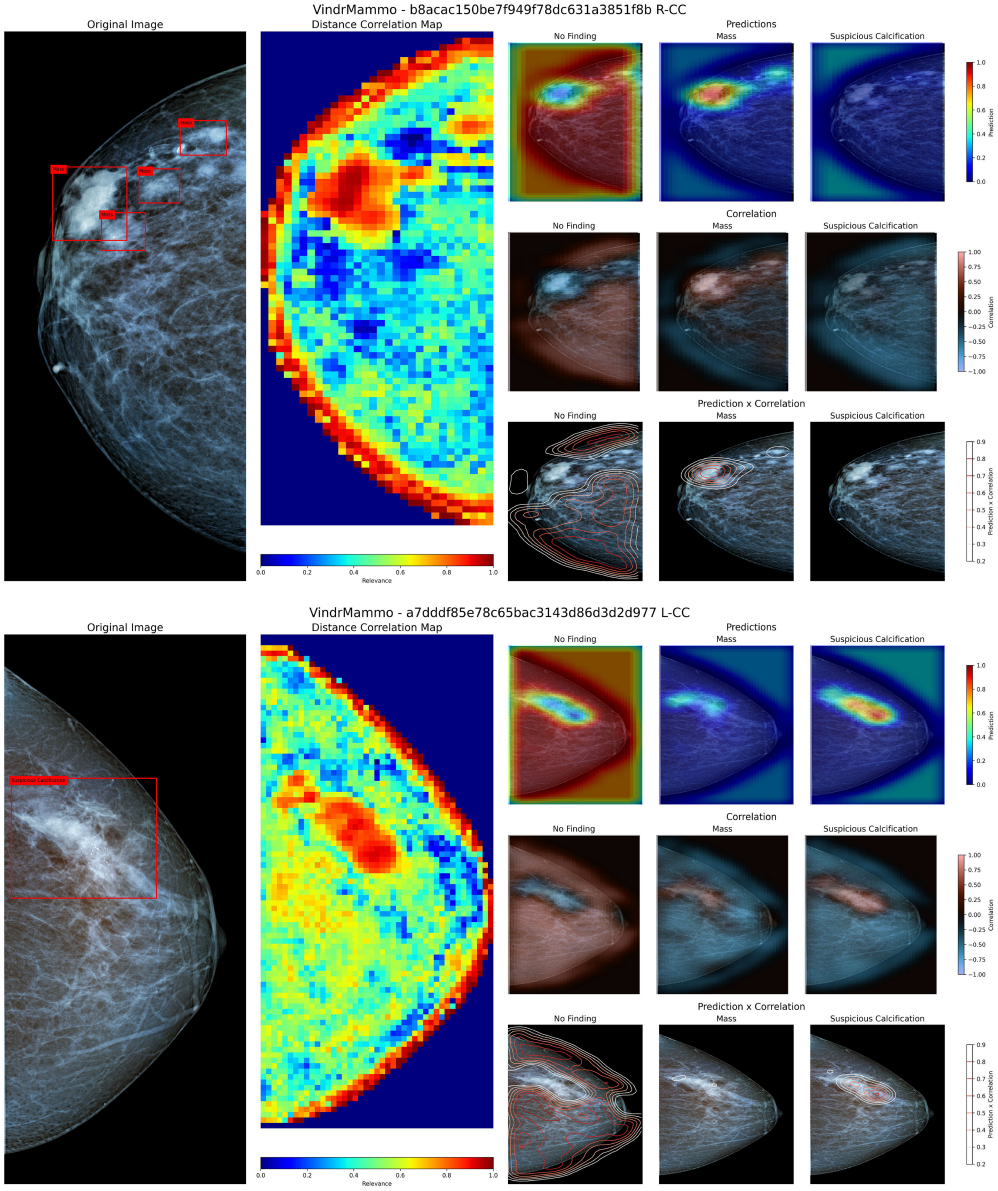
Visualization of the original image and annotated bounding boxes, distance correlation map, and sets of prediction, correlation and product heatmaps for the labels “No Finding”, “Mass” and “Suspicious Calcification” for two mammography images from the VinDr-Mammo dataset. The superior panel contains a group of masses in the upper region, while the inferior panel contains a large region labeled as containing suspicious calcifications.

**Figure 5** presents two examples from the VinDr-Mammo test set containing a group of masses (4a) and an exam showing a large area containing calcifications, evaluated using our method to visualize the identified regions containing these findings.

**Figure 5** superior panel contains a group of four labeled masses within a close region of the upper third of the breast. When inspecting the Distance Correlation map, the region containing these masses shows a high correlation value within the neighboring area. According to the model, this region contains more relevant information for its prediction than the rest of the image. According to the model’s knowledge, when inspecting both the prediction and correlation heatmaps, the region containing these masses shows a high probability of their presence and a positive correlation to that particular class. When combining the predicted values and the correlation map, we can coarsely delineate the region where these masses are located, allowing us to demarcate more precisely where these findings are present. In the case of **Figure 5** inferior panel, the presented view shows a large region labeled as containing suspicious calcifications. Using the proposed method, the combination of prediction and correlation maps delineates the area where these calcifications are located compared to the original bounding box. However, in this particular case, the model identifies a small region within the borders of the calcification as containing masses, albeit with a low probability of occurrence. Upon closer inspection, this misclassification may arise from the similarity to a mass-like structure with poorly defined borders, as both masses and calcifications appear in conjunction and share similar areas in the training dataset (52, 53).

### 4.2 Evaluation using Chilean patients’ mammography images

**Figure 6:**
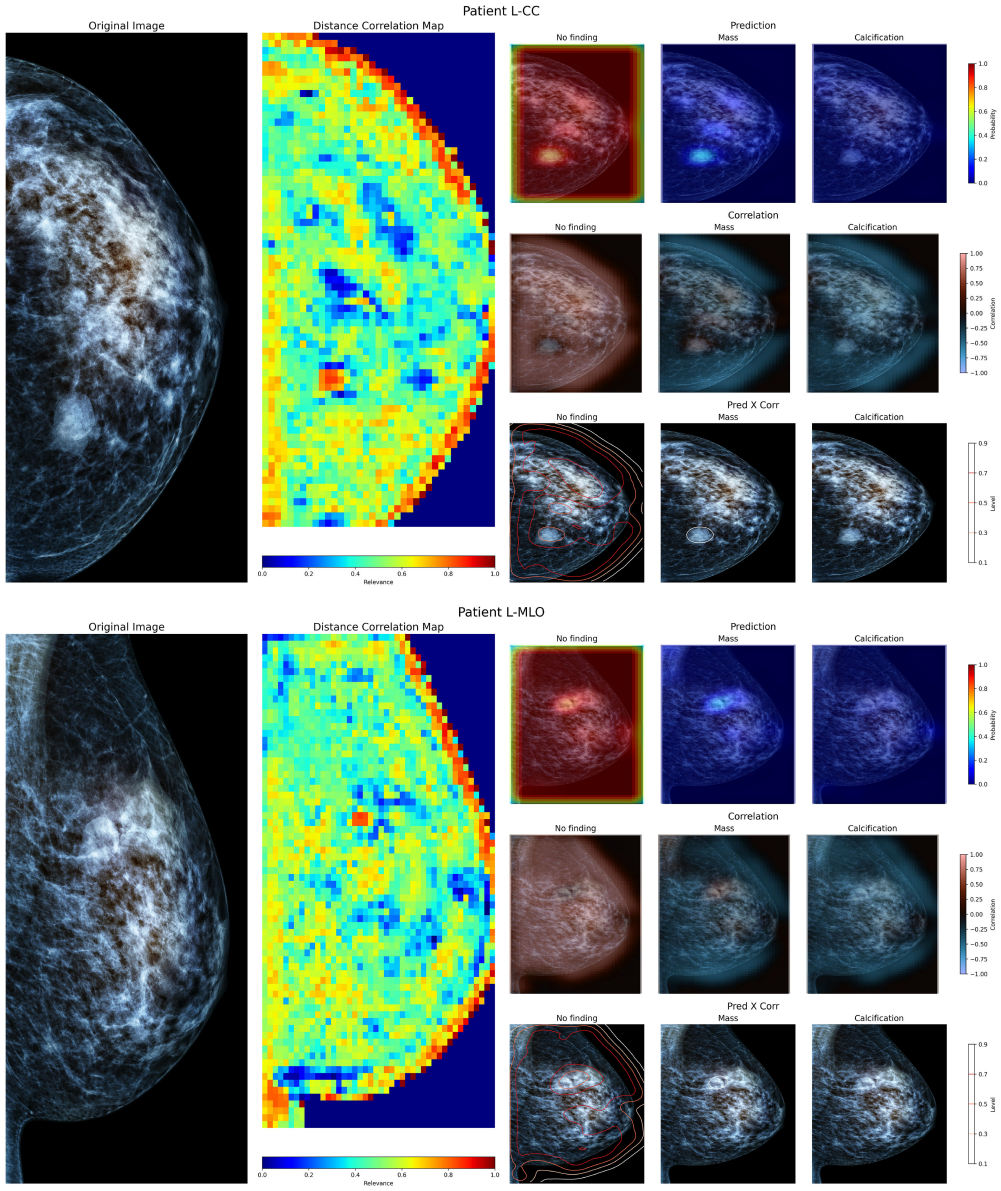
Visualization of the left breast from a patient of a local hospital, containing a defined mass within. The superior view shows the Cranio-Caudal view, whereas the inferior view shows the Medial-Lateral Oblique view of the same breast.

To evaluate the performance of the proposed method with local examinations, the model was applied to a set of mammography images from Chilean patients obtained from screening procedures conducted at a local hospital in Chile. Using the available exam report, the general location of masses present within was identified. **Figure 6** shows both cranio-caudal (**Figure 6** superior panel) and medial-lateral oblique (**Figure 6** inferior panel) views of the left breast from a patient. The report from this patient describes a mass on the left breast, located at the posterior third of the left upper inner quadrant, measuring 23mm in size. In both views, the mass is visible within the described region. On the Distance map, the demarcated region is identified as containing relevant information, albeit limited in size compared to the neighboring tissue, as neighboring windows start to include more normal tissue. When inspected using the prediction maps, the region shows a higher probability of a mass lesion’s presence on both views, but with a low confidence level. The correlation maps indicate a limited positive correlation between these regions and their neighboring areas. When combining both model prediction and class correlation, the detected mass is then delineated on both views. Showing that, despite the differences in image source, the proposed model has a positive response to a present lesion, and can identify the general location where masses and calcifications are present.

### 4.3 Sensitivity to number of masks in correlation

**Figure 7:**
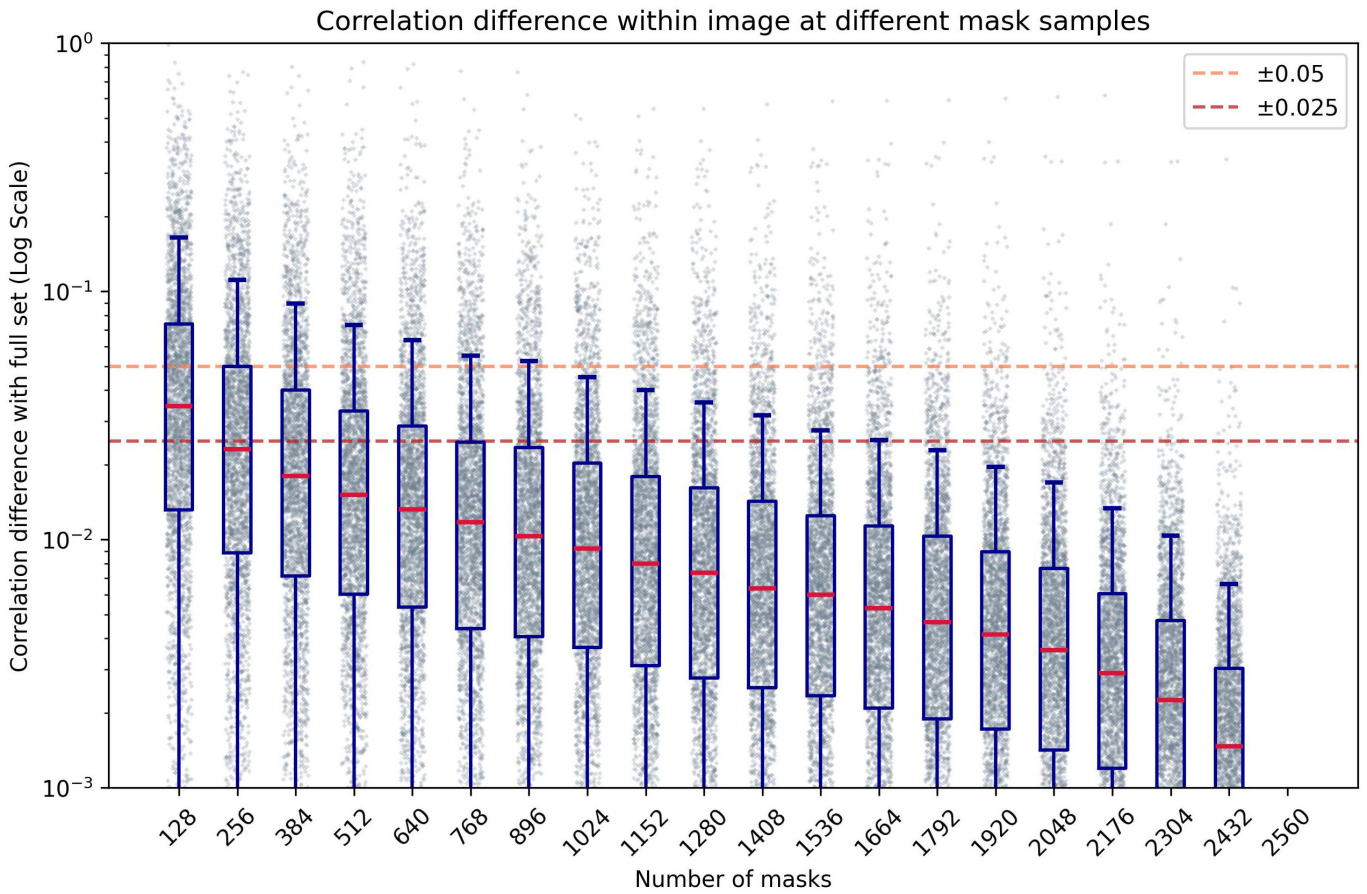
Box-plot of the absolute difference of correlation within an image compared to a high number of masks (n=2560), at different numbers of masks per window, in logarithmic scale.

**Figure 7** shows the effect of mask density on the correlation evaluation when compared to an arbitrarily high number of masks (2560). As the number of sampled masks decreases, the correlation error increases as expected. Using at least 256 masks per window, the mean correlation error from all windows remains below ±0.025. Using fewer masks results in less reliable values, as there are fewer combinations of features on each window to compare, adding bias to the interpretation of which areas within the evaluated window are more relevant to the resulting prediction. Conversely, using more masks increases the number of combinations of occluded regions, resulting in a more robust measurement of the linear relations of features and predictions. This introduces a trade-off between evaluation speed and correlation precision. While fewer masks can improve evaluation speed, using a large number ensures more reliable results, which is crucial for robust model interpretability.

### 4.4 Accuracy of location using pointing game metric

**Table 4** shows the accuracy for each label when evaluated using the “*Pointing Game*” strategy on the prediction and correlation heatmaps. Some images can contain multiple lesions of the same label, so these cases were counted as a single hit.

**Table 4:** Accuracy of “Pointing Game” evaluation of prediction and correlation maps compared to labeled bounding boxes.

Finding	Prediction Map (> 25%)	Prediction Map (> 50%)	Correlation Map	Support
Mass	0.5205	0.2654	0.4201	219
Suspicious Calcification	0.7714	0.5714	0.7238	105
Asymmetries	0.2692	0.0384	0.3462	78
Architectural Distortion	0.0000	0.0000	0.0000	24
Suspicious Lymph Node	0.4000	0.2000	0.0000	10
Skin Thickening	0.5833	0.4166	0.4167	12
Retractions	0.0000	0.0000	0.0000	8
Weighted Mean Accuracy	0.6358	0.3613	0.5602	456

**Figure 8:**
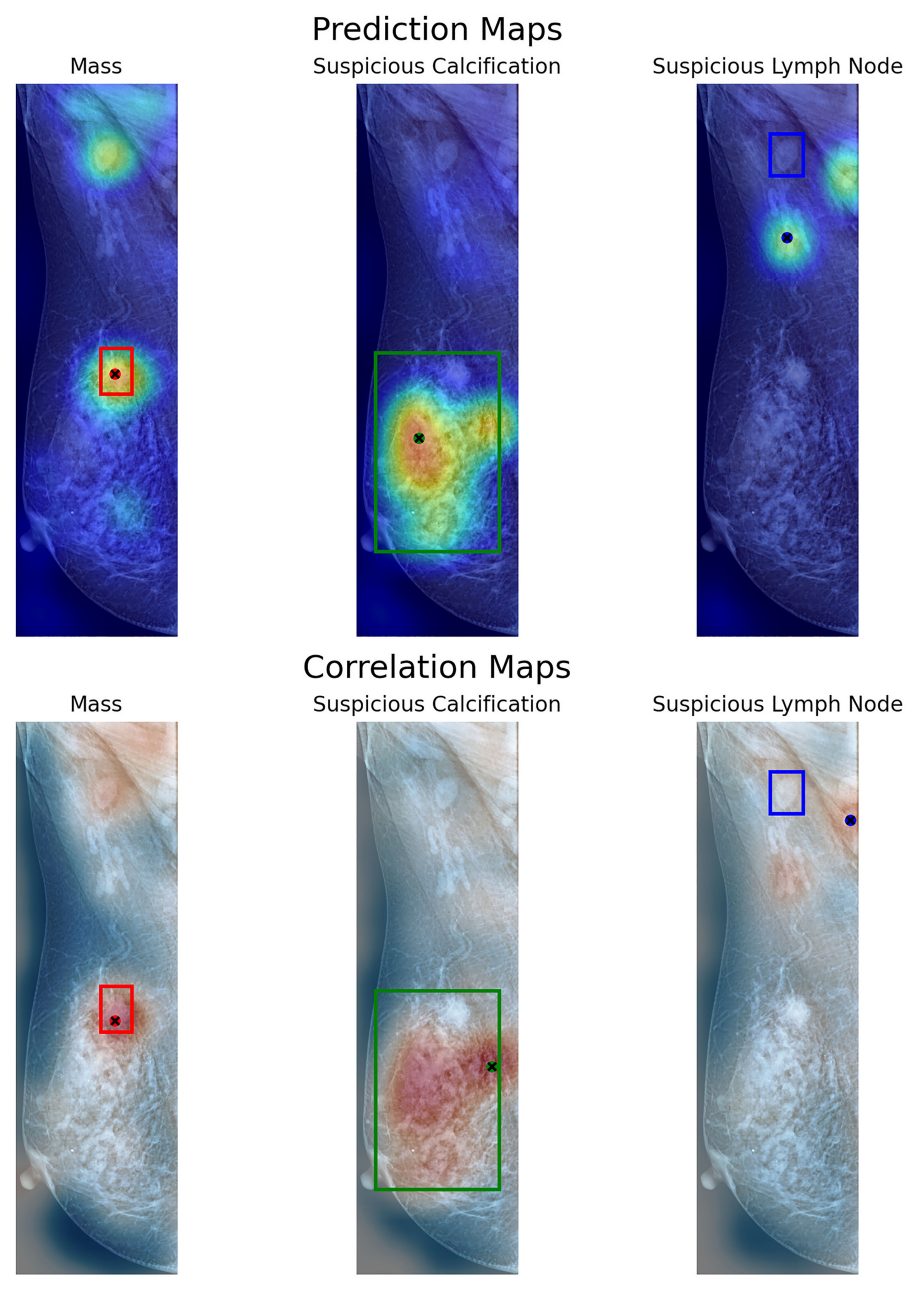
Visualization of prediction maps (above) and correlation maps (below) of three labels present on a VinDr-Mammo image with three annotated findings. Each box shows the labeled area as containing a particular finding. With a dot signaling the location of the maximum value of the map in that particular label.

Using the prediction map, the model achieves a weighted mean accuracy of 0.6358 with a detection threshold of 25%, whereas using a higher threshold of 50%, our model reaches 0.3613. As each window is weighted by its neighbors, using a higher threshold reduces the probability of detection when evaluated globally. In particular, the proposed method performed well at both threshold levels when identifying calcifications. Achieving a pointing game accuracy of 0.7714 and 0.5714 respectively at 25 and 50%. This indicates that the proposed model can locate the general region where calcifications are present, regardless of their size, when evaluated globally. In the case of masses, the prediction maps reach 0.5205 and 0.2654 at these thresholds. Using the correlation map, our model achieves a weighted mean accuracy of 0.5602, with similar results for masses (0.4201) and calcifications (0.7238). This shows that the measured correlation within each window can help more confidently locate the presence of lesions on a global mammography image. While somewhat inaccurate in some cases, our method can locate these lesions on most images using either method, as shown in our example from **Figure 8**. Notwithstanding, in the case of Skin Thickening, both prediction (at the threshold of 25%) and correlation maps achieve an accuracy of 0.5833 and 0.4167, respectively. This suggests that, at least for this particular finding, our model can effectively locate these when inspected globally. The model recognizes these findings as similar to its internal knowledge, despite the limitations imposed by the limited availability of samples.

## 5 Discussion

Saliency maps often provide information on the general location of image regions most influential to the output of DL models. However, their reliability is often limited by the model’s resolution, sensitivity to perturbations, and inherent limitations in identifying subtle features, in the context of medical imaging (54). Most existing approaches in the literature rely on global saliency extraction from the full mammography image (27, 32), which can hide the contribution of smaller lesions.

Grad-CAM remains one of the most widely used methods for visual explanations. Despite its popularity, its tendency to generalize over broad regions limits its effectiveness on high-resolution domains. In the case of mammography, where smaller lesions may critically influence diagnostic outcome, the provided explanation often fails to adequately explain their relation to the resulting output. Furthermore, previous work has shown low overlap to relevant features, compared to other interpretable methods (30).

In contrast, approaches that focuses on local information and their relation to elements similar to the target lesions have shown more alignment with clinical findings. Case-based interpretability methods have demonstrated the potential to improve radiologists’ decision-making, offering more intuitive insights compared to traditional gradient-based saliency maps (31).

Several recent studies have proposed explainability methods for visual attribution in breast imaging. Cerekci et al. (55) conducted a quantitative evaluation of saliency-based XAI methods, employing the “Pointing Game” strategy to assess the precision of their resulting explanation maps. They report a value of 41% for the detection of masses in mammography images using Grad-CAM, 30% with Grad-CAM++, and 35% with Eigen-CAM. By contrast, CorRELAX achieves 52.05% using the prediction map at a low-acceptance threshold, and 42.01% using the correlation map for the same task. Demonstrating competitive performance relative to gradient-based saliency methods. Nonetheless, some key methodological differences between both methods should be noted. First, Cerekci et al. method focuses solely on mass detection, while CorRELAX handles multi-label classification across different lesion types. Second, their analysis was performed on down-sampled mammograms, resized to 512 × 512 pixels, which may compress small-sized lesions that could be present, limiting their detection. While CorRELAX leverages high-resolution local windows and reconstructs prediction maps from overlapping patches, preserving spatial detail and improving sensitivity to smaller findings.

Gerbasi et al. (34) proposed a pipeline involving patch-based analysis for microcalcification segmentation and malignancy classification. Their method achieved strong quantitative results, reporting an IoU of 0.74 and an AUROC of 0.95 for detection of calcification clusters. However, their use of Grad-CAM and SHAP for explanation was limited to attributing malignancy to the identified clusters. And restricted to a single type of lesion, as with the case of the previous study.

Prinzi et al. (37) recently introduced a correlation-based method linking CNN features to radiomic descriptors in ultrasound breast images. Their approach addresses some of the limitations of saliency map explanations, specifically their consistency and extensibility in extracting global information. While their method differs from ours in modality and focus, it opens future opportunities for integrating radiomic interpretability into CorRELAX, potentially improving clinical robustness.

Despite the promising results, CorRELAX faces several limitations. First, the current experiment is constrained by the availability of labeled examples for less-represented findings such as asymmetries, lymph nodes, and architectural distortions. Most public mammography datasets only provide annotations for masses and calcifications, which limits their generalizability for smaller or less common lesions. Expanding annotated dataset could improve detection performance and increase clinical applicability.

Although the presented analysis confirmed that the resulting correlation metric is robust to the number of masks used, the resulting explanations remain dependent on the masking strategy and occlusion configuration. A more thorough analysis of these parameters could improve stability and efficiency of the explanation process, enabling real-time applicability.

Future work will also explore the application of CorRELAX to other medical imaging contexts, such as brain imaging (56), to evaluate how learned features correlate with radiomic information across different modalities. Additionally, a deeper integration of radiomic descriptors into the correlation analysis in mammography could further enhance the semantic richness of the provided explanations.

## 6 Conclusion

In this work, we presented CorRELAX, an algorithm for feature attribution analysis designed to measure the correlation between a deep learning model’s internal feature representation vectors and the resulting prediction from local regions using high-resolution mammography images. This method uses a deep CNN model trained to classify clinically relevant lesions in mammography images using fixed-sized sliding windows. The developed model can accurately classify normal tissue, masses, and suspicious calcification with a reported F1-Score of 0.9901, 0.7372, and 0.8402, respectively. Evaluating the global mammography image, our resulting correlation maps enable us to identify regions within the image that the model considers more relevant to the presence of specific findings. Reporting on the certainty of the model’s prediction when combined with the global predictions resulting from the model’s output. This method could provide new insights into the automatic identification and location of small pathological findings present within the breast when applied at early screening, before biopsy. Allowing the improvement of diagnostic evaluation times and giving more information to the radiologist for a more complete assessment of the risk of breast cancer.

## Data Availability

Publicly available datasets were analyzed in this study. This data can be found here: https://www.physionet.org/content/vindr-mammo/1.0.0/.
